# HUMAN AGING INTERVENTIONS

**DOI:** 10.1093/geroni/igac059.387

**Published:** 2022-12-20

**Authors:** Daniel Parker

## Abstract

Biological aging is the greatest risk factor for multiple non-communicable diseases affecting multiple organ systems. Evidence from animal studies suggest that the rate of biological aging is modifiable and that slowing the rate of biological aging may decrease the incidence of age-related morbidity and mortality. Based on these findings, several approaches are currently being studied to slow the rate of biological aging in humans. This symposium features internationally renowned aging research scientists whose work focuses on clinical interventions to modify biological aging in humans. We will hear from Rajagopal Sekhar from the Baylor College of Medicine who will present his research on “Improving oxidative stress, mitochondrial dysfunction, inflammaging, aging hallmarks and muscle strength in older adults: the novel role of GlyNAC and the 'Power of 3'”; Jamie Justice from the Wake Forest School of Medicine will present her work on “Response to dietary restriction interventions in older adults: biomarkers of cellular senescence and biological aging”; Reem Waziry from the Columbia University Mailman School of Public Health will present her work “Does Caloric Restriction Slow the Process of Biological Aging in Non-Obese Healthy Adults? Evidence from the CALERIE™ Trial”; and Daniel Parker from the Duke University School of Medicine will present his work “Does APOE genotype moderate the impact of diet modification and exercise training on age-related outcomes?”. Attendees will learn about recent advances in interventions targeting human aging.

